# An insight into planarian regeneration

**DOI:** 10.1111/cpr.13276

**Published:** 2022-07-10

**Authors:** Xin‐Yang Ge, Xiao Han, Yong‐Liang Zhao, Guan‐Shen Cui, Yun‐Gui Yang

**Affiliations:** ^1^ CAS Key Laboratory of Genomic and Precision Medicine, Collaborative Innovation Center of Genetics and Development, College of Future Technology Beijing Institute of Genomics, Chinese Academy of Sciences Beijing China; ^2^ Sino‐Danish College University of Chinese Academy of Sciences Beijing China; ^3^ China National Center for Bioinformation Beijing China; ^4^ Center for Reproductive Medicine, Henan Key Laboratory of Reproduction and Genetics The First Affiliated Hospital of Zhengzhou University Zhengzhou China

## Abstract

**Background:**

Planarian has attracted increasing attentions in the regeneration field for its usefulness as an important biological model organism attributing to its strong regeneration ability. Both the complexity of multiple regulatory networks and their coordinate functions contribute to the maintenance of normal cellular homeostasis and the process of regeneration in planarian. The polarity, size, location and number of regeneration tissues are regulated by diverse mechanisms. In this review we summarize the recent advances about the importance genetic and molecular mechanisms for regeneration control on various tissues in planarian.

**Methods:**

A comprehensive literature search of original articles published in recent years was performed in regards to the molecular mechanism of each cell types during the planarian regeneration, including neoblast, nerve system, eye spot, excretory system and epidermal.

**Results:**

Available molecular mechanisms gave us an overview of regeneration process in every tissue. The sense of injuries and initiation of regeneration is regulated by diverse genes like follistatin and ERK signaling. The Neoblasts differentiate into tissue progenitors under the regulation of genes such as egfr‐3. The regeneration polarity is controlled by Wnt pathway, BMP pathway and bioelectric signals. The neoblast within the blastema differentiate into desired cell types and regenerate the missing tissues. Those tissue specific genes regulate the tissue progenitor cells to differentiate into desired cell types to complete the regeneration process.

**Conclusion:**

All tissue types in planarian participate in the regeneration process regulated by distinct molecular factors and cellular signaling pathways. The neoblasts play vital roles in tissue regeneration and morphology maintenance. These studies provide new insights into the molecular mechanisms for regulating planarian regeneration.

## Graphical abstract

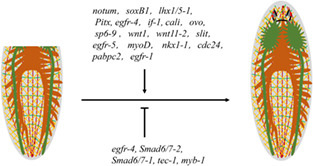

## 1 INTRODUCTION

Considering the great threat to animal survival posed by injury‐induced loss of organs, regeneration is always an attracting topic in the field of medicine. According to the previous studies, the regeneration process occurs extensively in certain kinds of animals. For example, researchers have uncovered the regeneration ability of limb (axolotl), heart (zebrafish) and liver (human) after injury.^1^, ^2^, ^3^ However, the exact biological mechanisms underlying regeneration are currently poorly understood and need to be further elucidated.

Planarians are flatworms capable of regenerating any missing body region, making it an ideal model to study the in‐depth mechanisms of regeneration. The powerful regenerative ability of planarians benefits from a proliferative cell population that contains pluripotent stem cells, also called neoblast. Neoblasts widely distributed throughout the body, and account for 25%–30% of the body cells in the whole worms.^4^ When injury happened, neoblasts near the wound migrate to the injury site and differentiate into tissue specific progenitor cells, then form a transparent tissue called blastema.^5^, ^6^, ^7^ The blastema will express PCGs (positional control genes) for polarity determination.^8^ The specification of the blastema and the subsequent formation of local signalling centres allow for the growth and patterning of the blastema, leading to the highly organized regeneration of lost body parts.^9^, ^10^ The migration, oriented differentiation and self‐renewal of neoblast during planaria regeneration are regulated by a series of biological processes like cell cycle, apoptosis, proliferation and so on. Recent studies have shown that planaria can regenerate all tissue parts. Various signalling pathways and molecular mechanisms play important roles in the regeneration process of planarians. It is these organized specific regulation that enables planarians to accurately regenerate appropriate tissues after injury.

In this review, we first discuss the biological background of planarians, and then summarize the most recent findings about the critical regulatory factors and the underlying genetic and molecular mechanisms for the process of regeneration within every tissues of planarian.

## 2 NEOBLAST: THE FOUNTAIN OF REGENERATION

The regenerative ability of planarians is mainly related to neoblasts. Therefore, it is of great significance to reveal how this cell type is involved in the regeneration process. As neoblasts are distributed throughout the body, the key to the above puzzle is to clarify the mechanisms for their directional differentiation into other cell types. First, planarian stem cells are considered to be heterogenous cells with a high nucleo‐cytoplasmic ratio, with a cell diameter of about 5–10 μm. At the same time, a commonly recognized characteristic of planarian stem cells is that the cytoplasm contains more chromatoid bodies, which enable the neoblasts to respond quickly to external stimulis.^11^ In planarians, all dividing cells except sperm and eggs are considered as neoblasts.^4^ Because of this characteristic, stem cells are sensitive to X‐rays and gamma rays^12^, ^13^ which can stall cell division. The application of labelling and staining techniques also enables us to identify these stem cells. By staining the dividing cells with BrdU, the spatial and temporal distribution of stem cells can be clearly unvealed,^14^ and In situ hybridization can locate the neoblasts with specific marker genes like *smedwi‐1*, *tgs‐1* and *tspan‐1*.^15^, ^16^, ^17^

Neoblasts with dividing capability have significant role in regeneration, but not all neoblasts have the ability to differentiate into other cell types. Wagner et al. identified a group of neoblasts called cNeoblast (Clonogenic Neoblasts), cNeoblast can differentiate into all other tissues. If a cNeoblast was transplanted into a planarian that had been irradiated with X‐rays to ablate all existing neoblast, the planarian could survive and regenerate neurons, muscles, protonephridia, intestines and all other tissues.^18^ cNeoblast can be isolated by fluorescence‐activated cell sorting using surface protein marker TSPAN‐1.^16^ By single‐cell sequencing analysis, the researchers divided the planarian neoblasts into 12 different subpopulations, in which NB2 was the most pluripotent subpopulation. NB2 can differentiate into various progenitors including epidermal progenitor (NB3), muscle progenitor (NB4), gut progenitor (NB5), pharynx progenitor (NB7), protonephridia (NB9) and neural progenitor (NB11), which then differentiate into specific cell types under the regulation of different genes.^16^ Upon injury, neoblasts in planarians can response immediately with two peaks of mitotic cell numbers (6 and 48 h post‐amputation) in mitotic cell in numbers. The first peak of neoblasts proliferation occurs at 6 h, involving a wide range of neoblasts distributed throughout the body and being responsible for initiating the wound healing and regeneration process. The second peak occurs specifically following major injuries (amputation) and is biased towards wound sites.^19^ Following the tissue loss, there are two peaks of apoptosis for tissue remodelling. Cell death (TUNEL^+^ cells) occurred proximally at any wound within 4 h of injury, while a second sustained phase of elevated cell death involved in morphallaxis is specifically associated with tissue loss injury. These proliferative and cell death responses specific to missing tissue injury at 48–72 h after amputation, are specific to missing tissue injury, a striking features of planarian regeneration.^20^, ^21^, ^22^

Neoblast repopulation can be achieved from even a single pluripotent stem cell in planarians. Proliferation of neoblasts involves symmetric and asymmetric division.^23^, ^24^ In the asymmetric division, neoblast divides into one self‐renewing cell and one differentiated cell. This process is regulated by the Egf signalling pathway. EGFR‐3 as the membrane surface of neoblasts is asymmetrically distributed when division initiate. Cell with more EGFR‐3 protein turn out to be self‐renewing cells, while the other cells without EGFR‐3 protein differentiates into other cell types under the regulation of different signalling pathways. Interestingly, inhibition of *egfr‐3* results in the failure of cell division suggesting that *egfr‐3* regulates asymmetric cell division in neoblast repopulation.^25^ Neoblasts is essential for planarians, but when and where the neoblasts differentiate in the process of regeneration and the mechanisms of regulating the termination of neoblasts differentiation are still a blur. Accordingly, the spatial technologies and other aspects of investigations is needed to elucidate these problems.

## 3 GENERIC WOUND RESPONSE: HEALING OR REGENERATION?

How does the planarian distinguish the severity of injuries and determine the destinations of repair process, only requires wound healing or need to further regenerate missing tissue? What are the molecular mechanisms differentially controlling these process? Irrespective of healing or regeneration, more than 200 genes are activated in planarians after injury, but only amputation will result in the induction of missing‐tissue response (MTR).^26^ Gavino et al. found that *Smed‐follistatin* is a damage sensory gene during planarian regeneration. Moreover, *follistatin* is only required for responding to tissue loss following injury, and does not expressed during homeostatic process, suggesting that *follistatin* can induce MTR.^26^ MTR can regulate the speed of regeneration. If the *follistatin* is blocked, planarians cannot regenerate successfully. The *follistatin* RNA interference (RNAi) targeted tail fragments will not be able to regenerate the lost tissue and no longer express PCGs, so the anterior–posterior (A/P) axis cannot be re‐established. The *follistatin* negatively regulates *wnt1*, thus affecting head regeneration.^27^ Owlarn et al. showed that virtually all injuries have the ability to induce regeneration, but regeneration only occurs when planarian tissues are lost or PCGs dysfunction, such as the Wnt signalling pathway.^28^ They found that the expression of ERK signalling pathway increased significantly in a short time after injury, and the regeneration of planarians could not be carried out after disturbing any components of ERK signalling pathway. When the inhibition of ERK signalling pathway was removed, the regeneration of planarians can restart, indicating crucial roles of ERK signalling in initiating the regeneration process.^29^

## 4 ENVIRONMENTAL FACTORS INFLUENCE REGENERATION

The regeneration of planarians is also affected by environmental factors, such as temperature, gravity, reactive oxygen species and other factors. Researchers found that the optimal culturing temperature of planaria is from 19 to 25°C.^30^ The study by Hammoudi et al. showed that the regeneration speed of planarian was the highest at 26°C. The planarian *Schmidtea mediterranea* would not survive between 30 and 32°C. However, there was no significant difference in migration and feeding behaviour of planarians at temperatures between 19 and 28°C. They also demonstrated that RNAi remained effective at 26 and 28°C by feeding dsRNA. Interestingly, the planarians reared at 19°C were able to completely clear *Staphylococcus aureus* within 6 days, while this clearing duration was shortened to less than 3 days cultured between 26 and 28°C, indicating that the immune response was intensified during 26–28°C.^31^ Therefore, there also exist commensal bacteria in planarians, previous studies have shown that one planarian can harbour 42 distinct bacterial operational taxonomic units (OTUs), and the bacteria will delay the regeneration process of planarians by producing indole.^32^ Michael Levin lab found that *Aquitalea sp. FJL05* is an endogenous commensal bacterium of *Dugesia japonica* planarian. *Aquitalea sp. FJL05* can influence A/P axial and positional patterning during regeneration, which will result in two‐headed animals.^33^ Since there are many bacteria found in planarians, but not all the functions of these bacteria have been studies. In this case, it would be interesting to explore the possible effect of these bacteria on the planarian regeneration.

Gravity could also be an important factor regulating regeneration. Teresa et al. from the Netherlands conducted their experiments at the European Space Research and Technology Centre in Noordwijk and investigated the effects of microgravity on the regeneration of the planarian *Schmidtea mediterranea*. They used random positioning machine (RPMS) or the large diameter centrifuge (LDC) to simulate microgravity at speeds of 60°/s and 10°/s. The results showed that RPMS of 60°/s resulted in the death of the planarians, while those loaded with RPMS of 10°/s showed normal regeneration (normal eyes and central nervous system, similar mitotic activity). In addition, the injured planarians do not die soon under the condition of the 60°/s RPM machine but after having regenerated the main structures. They hypothesized that planarians had rheostatic vessels in their heads that could sense the flow of water at 60°/s RPM and induce death as their entire head were regenerated. They also studied the effect of hyper‐gravity on the regeneration of the planarian *Schmidtea mediterranea*, by simulating a high gravity at 3, 4 and 8 g using a LDC. Under 3 and 4 g hyper‐gravities, planarians could regenerate the missing tissue, but the proliferation rate decreased. At 8 g, only the larger trunk, but not the smaller fragment of planarians, could regenerate the missing part.^34^, ^35^

Pirotte et al. studied the effect of reactive oxygen species (ROS) on the regeneration of the *Schmidtea mediterranea*, and claimed that ROS is an early signal to induce tissue regeneration. They truncated planarian into three fragments at the pre‐ and post‐pharynx levels to study the effect of ROS on regeneration of different body parts. They found that ROS bursts within minutes after amputation, and its production was independent of wound localization, while signals that initiate the regeneration process will only be induced at least 24 h after amputation. Inhibiting ROS production resulted in the failure of three segments to regenerate lost parts, and meanwhile, reduced ROS restricted regeneration of cephalic ganglia and ectopic neuronal cells. Interfering with ROS production did not affect neoblasts proliferation, but constrained the differentiation of neoblasts into desired cell types.^36^ These suggests that environmental factors are crucial in the process of regeneration, and missing any of these factors will result in the failure of regeneration. Investigations of environment factors will be an indicator for human living conditions. For example, studies on the impacts of different kind of pollution on regeneration will help us understand a lot diseases and give us a hint on environment protection.

## 5 IMPACTS OF PHYSIOLOGICAL FACTORS ON REGENERATION

Cilia, which controls the movement of planarians, is an important tissue of planarians. They located on the ventral side of epidermis, and ciliary rootlets are globally polarized. Levin et al. established a framework for the alignment of cellular polarity vectors relative to planarian body plan landmarks. In their article, they found that ciliary rootlets can be regard as the indicator of epidermal polarity, which is defined by head, tail and the body margin.^37^ Cellular polarity is also a crucial factor for regulating planarian regeneration. Levin et al. created double head planarians to demonstrate the flexibility of the body polarity. By visualizing the orientation of cilia beat and cilia‐driven flow in living planarians, they found that ciliary beat reorientated within several weeks to match the new axial body plan, and the progresses is independent from cilia beating.^38^

Calcium (Ca^2+^) channel play an important role in depolarization‐induced contractions of muscle,^39^ which is indispensable to flatworm neuromuscular physiology. Knockdown of the subunit of Ca^2+^ channel will lead to loss of mobility.^40^ PCGs are mainly expressed in muscle, indicating the potential correlation between Ca^2+^ channel and A/P axis. Praziquantel, which targets Ca^2+^ channel, will lead to ectopic head in flatworms, suggesting the role of Ca^2+^ channel in regulating stem cell differentiation and A/P axis patterning during regeneration.^41^ Understanding the function of bioelectric, neurotransmitter and cell polarity during regeneration will link the physiological factors with molecular mechanism, which will help us to better understand the complex network underlying regeneration.

## 6 ANTERIOR AND POSTERIOR POLARITY DICTATES PLANARIAN PATTERNING DURING REGENERATION

When planarian is injured, such as the head or tail amputation, signalling pathways guiding the error‐free regeneration, that is the right tissues and organs in the right place, remains to be elucidated. Studies have shown that the Wnt signalling pathway is the key regulator of the polarity in planarians. *β‐catenin‐1* RNAi will cause planarians to generate heads at any wounded site, resulting ectopic heads.^42^, ^43^, ^44^ Knockdown of *wntless*, *wnt1*, *teashirt* and *dishevelle* genes will result in the planarian to regenerate its head at the tail place and grow multiple heads throughout the body.^44^, ^45^, ^46^ Conversely, silencing the genes that are known as the inhibitor of the Wnt signalling pathway produces ectopic tail regeneration. Additionally, the Wnt‐related genes are all distributed dependently along the A/P axis of planarian, and are specifically expressed in different regions. For example, genes in the Wnt signalling pathway, including *wntP‐2* (*wnt11‐5*), *wnt11‐1*, *wnt11‐2*, *fz4‐1* and *wnt1* are genes specifically expressed in the tail,^47^ whereas the Wnt pathway inhibiting genes, such as *sFRP‐1*, *sFRP‐2*, *foxD*, *fz5/8‐3* and *notum*, are only expressed in the head region of planarian.^48^, ^49^ These findings illustrate the critical roles of the Wnt signalling pathway in regulating the polarity of planarians, pointing to the significance of Wnt functions in other species. Meanwhile, the expression of Wnt is regulating by hedgehog signalling, indicating the hh signalling is indispensable for A/P axis.^50^

Interestingly, studies have shown that bioelectric signals in the very early stage of regeneration modulate the re‐establishment of planarian A/P axis. Exploring the function of bioelectric signalling during regeneration will help us to understand the relationship between complex physiological patterns and molecular pathways. As early as 3 h post amputation (3 hpa), bioelectric signalling will participates in the A/P axis formation in planarians. In this case, depolarized the endogenous bioelectric at 3 hpa will create a double‐headed worm.^51^ Furthermore, the abnormality of A/P axis has is permanent effect even after briefly disturbed the endogenous bioelectrical networks is permanently,^52^ indicating that bioelectric physiology is essential to animal morphology during planarian regeneration.

In addition, Bmp signalling pathway has been demonstrated to regulates the dorsal‐ventral (D/V) axis. The absence of any component in the Bmp signalling pathway, such as *bmp4*, *smad1*, *smad4* or *tolloid*, can lead to abnormal D/V polarity.^53^, ^54^ Gavino and Reddien's team found that inhibition of Bmp signalling promotes the formation of ventral region, resulting in a planarian containing cilia on both dorsal and ventral sides.^55^ Cebria et al. have recently identified a planarian genes‐*slit* whose functions are critical for patterning and midline regeneration. *slit* is a secreted extracellular ligand of the Roundabout (Robo) receptor family, and *slit* knockdown results in cyclopic anterior regeneration and midline collapsed photoreceptors. Ectopic neural tissue also appeared in the midline in uninjured *Smed‐slit* (RNAi) animals with long term culturing, suggesting crucial roles of *slit* in the maintenance of normal homeostasis of CNS tissue.^56^ Also, Levin et al. use RNAi screening to validate that the Ft/Ds pathway oriented ciliary rootlets towards the body margins, which will affect the D/V axis.^37^

Reddien et al. found that when eliminating neoblast with irradiation, dramatic change of some PCGs still existed during regeneration, indicating that PCGs are not expressed in neoblasts. Several studies showed that the expression of most PCGs is restricted to a layer of subcutaneous cells around the neoblasts.^47^, ^57^, ^58^ Subsequently, single‐cell RNA sequencing on muscle cells from multiple regions along the planarian AP axis revealed that many additional genes exhibited regional expression in certain muscle tissues.^59^, ^60^ *wnt1* is activated and expressed at approximately 6 h after injury, and its knockdown causes posterior‐facing wounds to regenerate ectopic heads or fail to regenerate, suggesting that *wnt1* promotes posterior regeneration and inhibits anterior regeneration.^58^ *wntP‐2* is specifically expressed in the tail fragment, and has no expression in the head region after *wnt1* knockdown, but the expression of *wntP‐2* could be detected in the head of the irradiated planarian. While *wnt1* is expressed in the posterior pole of uninjured animals, *notum* is a conserved inhibitor of Wnt signalling pathway oppositely expressed in the anterior pole of the planarian head. *notum* RNAi animals regenerate the tail at the location of the head, suggesting that *notum* promotes anterior but inhibits posterior identity. In addition, *wnt1* and *notum* are also considered as ‘generic wound response’ genes.^9^, ^44^, ^47^, ^61^ In conclusion, Wnt signalling pathway regulate the A/P axis of planarian, and Bmp signalling pathway regulates the D/V axis. Since then, even countless researches explained the polarity definition, there still a lot mysteries exist such as how these genes and bioelectric signals coordinate with each other and what is the temporal and spatial expression of these genes? We think the studies based on crosstalk of different fields to reveal regeneration need to be carried out.

## 7 MECHANISM OF TISSUE‐SPECIFIC REGENERATION

Apart from the underlying molecular mechanisms and related environmental factors for neoblasts in modulating regeneration process, accumulating data have also shown the tissue‐specific regeneration mechanisms. The followings are the summarization of recent progress about the findings on each tissue.

### 7.1 Eye spot

**FIGURE 1:**
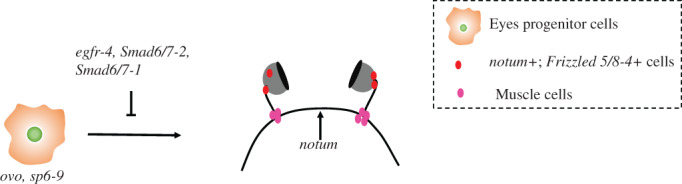
Overview of recent progresses on eye spot regeneration. *ovo* and *sp6‐9* specifically express in eye progenitor cells. Regeneration of eye spot is regulated by *egfr‐4*, *Smed‐smad6/7‐1* and *Smed‐smad6/7‐2*. *notum* directs the position of axon. Guidepost‐like cell, existing outside of visual system, acts like a landmark to guide the eye's regeneration.

Planarian eyes connect to the brain via axon bundles, thus are true brain eyes with high expression of homologous genes like *Otx*, *Sine oculis*, *ovo* and *eyes absence*.^62^, ^63^ Planarians have photoreceptor neurons and pigmented optic cup cells. Planarians' eye spots are important photoreceptors, and can fully regenerated after excision^62^ (Figure 1).

*egfr‐4* has been shown to play an important role in the regeneration process of planarian eye spot. Worms with *egfr‐4* silencing by RNAi showed much smaller regenerated eyes than the control group. In addition, the numbers of mature eye‐specific cell type including photoreceptor neurons and pigment optic cup cells were significantly reduced in *egfr‐4* RNAi worms, whereas the eyes progenitor cells which expressing specific markers *ovo* and *sp6‐9* had an increased number. These results suggest that *egfr‐4* is not required for the production of eye progenitor cells, but for their final differentiation (Figure 1), supporting the idea that the Egf pathway in eukaryotic cells may play a general role in regulating differentiation of progenitor cells.^64^

Recently, Scimone et al. found that the eye regeneration process of planarians is regulated by guidepost‐like cells.^65^ They found a rare subpopulation of muscle like cells (defined as guidepost‐like cells; expressed *notum* and *frizzled 5/8‐4*) concentrated at two precise anatomical sites and intimately associated with the photoreceptor axon. In the place of a bundle of visual axons project and fasciculate lies the first group of guidepost‐like cells. Also, near the choice point, where sorting of contralateral and ipsilateral axons occurs, lies the second group of cells. Those two groups of muscle like cells are formed during the regeneration of the neuron system, which are related to axonal projections. They also identified a group of *notum‐*expressing neurons which are located in adult anterior brain commissure, these neurons express *notum*. When the eyes were transplanted into the ectopic anatomical locations, *notum+*; *Frizzled 5/8‐4+* muscle like cells were undetectable. In addition, planarian eye lost regeneration ability after *ovo* (RNAi), while the muscle‐like cells can still be generated in the right place. Guidepost‐like cells are located at the crucial place in nervous system, where they form independently from photoreceptor axon, regulated by PCGs external regulatory mechanism (Figure 1). Therefore, it is hypothesized that guidepost‐like cells exist outside of visual system and are regulated by PCGs.^65^

Bone morphogenetic protein (BMP) pathway also plays a role in planarian eye spot regeneration. Studies have demonstrated the function of *smad1/5/8* and *smad4* in planarian D/V axis re‐establishment. *Smed‐smad6/7‐1* and *Smed‐smad6/7‐2* are the two inhibitory Smads (I‐smads). *smad6/7‐1* is expressed in parenchyma, while *Smad6/7‐2* expression can be detected in the central nervous system and eyes. Inhibition of either of these two genes will result in abnormal D/V axis. In homeostatic and amputated planarians, their knockdown could cause defective small and rounded eyes lacking anterior subpopulation of photoreceptor cells. The number of pigment cells in these knockdown planarians also decreased during the later stages of regeneration. In contrast, through reducing the inhibitory effect of *Smed‐BMP* (RNAi), planarians regenerated larger eyes with expanded subpopulation of photoreceptor. These results demonstrate that *Smad6/7‐2* and *Smed‐BMP* control the maintenance of anterior photoreceptor cell numbers in planarian^53^, ^54^ (Figure 1).

### 7.2 Central nervous system

The nervous system is distributed throughout the planarian whose central nervous system (CNS) consists of a pair of cephalic ganglia and ventral nerve cords which are comprised of a cortex of neuronal cell bodies surrounding a neurite‐filled neuropil.^66^, ^67^ The most typical cell types include serotonergic, GABAergic, dopaminergic, octopaminergic, cholinergic and glutaminergic neurons.^66^ Planaria head regeneration is a complex and intense process, which is initiated by the wound signalling after injury involving stem cells proliferation and migration to the head region to form a transparent tissue called blastema. The neoblast differentiates into neural system and eventually to regrow the head tissue. To further explain the molecular mechanism underly the CNS regeneration, we review some of newly discovered genes that essential to CNS regeneration and maintenance.

**FIGURE 2:**
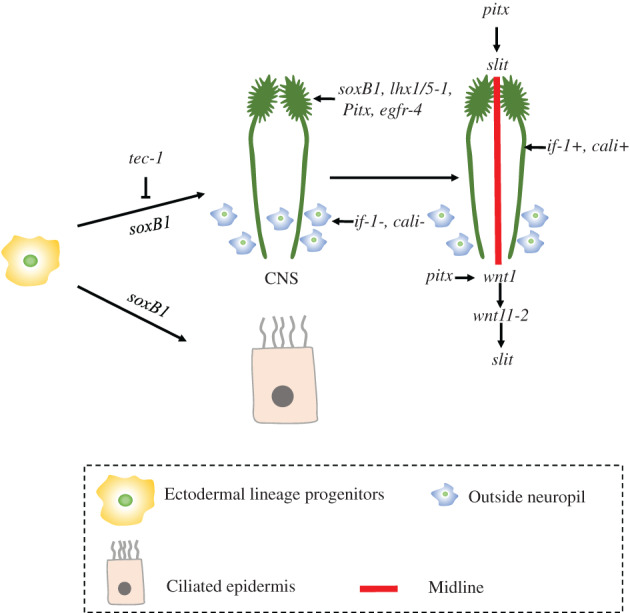
Recent progresses on central nervous system (CNS) regeneration. Ectodermal lineage progenitors direct the differentiation of epidermal and neuronal cells. Regeneration of CNS is regulated by *soxB1*, *lhx1/5‐1*, *pitx*, *egfr‐4* and so on. *pitx*, *slit*, *wnt1* and *wnt11‐2* construct the midline of CNS.

Recent studies showed that the CNS regeneration is negatively regulated by *tec‐1*, while the maintenance of CNS is related to *SoxB1*. The gene *tec‐1* is a negative regulator of neuron regeneration in planaria, with inhibitory role in the neurons regeneration of the brain. It was found that inhibition of *tec‐1* increased GABAergic, serotonergic, nociceptory *trpA+* neuron populations. The number of peripheral cholinergic *chat+* neurons, Cephalic *ppl‐1+* cells, serotonergic neurons and *dd17258+* neurons also showed an increase after *tec‐1* inhibition, Thus, *tec‐1* appears to negatively regulate the abundance of many types of neurons in the central and peripheral nervous systems except *ppl‐1+* cells whose abundance in neurons of the pharynx and photoreceptor neurons were not affected post *tec‐1* inhibition^68^ (Figure 2).

*SoxB1* gene is required for regeneration and maintenance of the planarian sensory neuron populations of the planarians. Inhibiting the activity of *SoxB1* or genes that regulated by *SoxB1* impairs sensory function and triggers seizure‐like movements. The brains size of *SoxB1‐2* (RNAi) planarian shrank overall. Single‐cell sequencing analysis showed that *SoxB1‐2* mainly functions in ectodermal lineage progenitors and directs the differentiation of epidermal and neuronal cells. These data implicate important role of *SoxB1‐2* activity in cells with sensory neuron function^69^(Figure 2).

Serotonin neurons in planarians are thought to be spatially restricted to the ventral side of the worms and their axons projections extend across the mediolateral body axis. *Smed‐lhx1/5‐1* and *Smed‐Pitx*, which are necessary for the maintenance and regeneration of serotonergic neurons in planarians (Figure 2). It was found that the marker genes *tph* and *aadc* were not expressed in serotonergic neurons when *lhx1/5‐1* or *Pitx* were eliminated by RNA interference. This indicates that *lhx1/5‐1* and *Pitx* were required for the maintenance of serotonergic neural identity in uninjured animals. *Pitx* is also required during the regeneration of the planarian midline.^70^

There are also many signalling pathways that regulate neural regeneration. *Smed‐egfr‐4* is the downstream target of *Smed‐egfr‐3* and *Smed‐egfr‐4* is mainly expressed in the brain and necessary for brain regeneration. *Smed‐nrg‐7* is a ligand of *Smed‐egfr‐3*. Knockdown of *Smed‐nrg‐7* shows the same phenotype as *semd‐egfr‐3*(RNAi). *Smed‐nrg‐7* is also expressed in the brain and regulates stem cell division and specification. *Smed‐egfr‐3* and *Smed‐nrg‐7* play an important role in neuron regeneration.^64^, ^71^

Reddien et al. found that hedgehog (hh) is expressed in medial cephalic ganglia neurons and its inhibition led to the expression of *filament‐1* (*if‐1*) and *calamari* (*cali*) express in unidentified non‐neural CNS cell type (Figure 2). This type of cells expresses homologues of astrocyte‐related genes involved in neurotransmitter uptake and metabolism, as well as extended processes covering regions of high synaptic concentration. It has been shown that the state of the glia is regulated by hh signalling.^72^ As neuron regeneration is an intricated process involving many regulatory processes. One single gene or pathway alone cannot direct the whole process. Transcriptomic sequencing analysis by Roberts et al. on the post‐pharyngeal amputated planarians, found that about 45% of the transcripts were up‐regulated during regeneration, including the aforementioned list of genes. RNAi screening on 326 up‐regulated genes reveal that 9.2% (30/326) of genes could result in defective neuron regeneration after knockdown. These genes regulate nerve regeneration through multiple pathways, for example specification of neuronal progenitors, and anterior polarity and neural patterning.^73^

### 7.3 Excretory system

**FIGURE 3:**
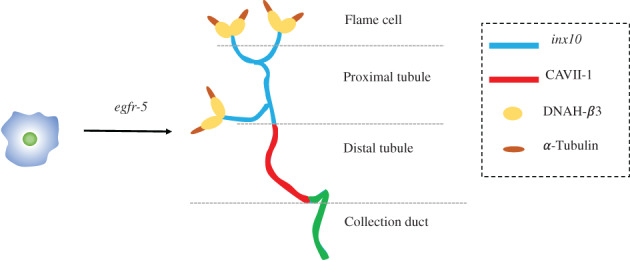
The maintenance and regeneration of the planarian excretory system are regulated by EGFR signalling. Right part: Molecular anatomy of protonephridia.

The excretory system of planarians consists of the protonephridia, which are branched organs widely distributed throughout the body. Protonephridia are composed of ciliated “flame cells” attached to long tubules or cilia that end on the surface of the planarians.^71^ Researchers have identified all genes from the planarian genome, which encode *solute carrier* (*slc*) transporter proteins being capable of transporting molecules in and out of protonephridia cells. A total of about 300 *slc* proteins were identified and their expression patterns were mapped in planarians by in situ hybridization. About 15% of these transporter genes were found to be expressed in the protonephridia.^74^ As discussed above, 6 members of EGFR family exist in planarians, and *egfr‐3* and *egfr‐4* regulates neoblast repopulation and eye spot regeneration, respectively. In support, *egfr‐5* was demonstrated to be involved in the morphological maintenance and regeneration of the excretory system of planarians. Its depletion reduced the regeneration ability of planarian with feature of the lost original morphology in the excretory systems, pointing to the significance of *egfr‐5* in flame cell maintenance and branching morphology^71^, ^75^ (Figure 3).

### 7.4 Muscle

**FIGURE 4:**
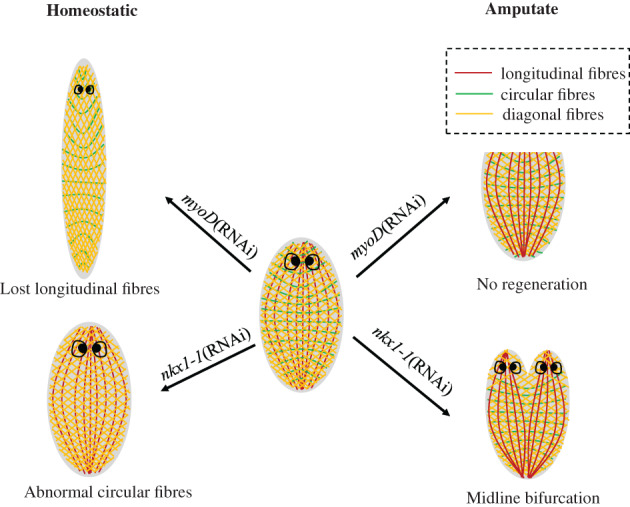
*myoD* is required for the formation of longitudinal fibres, while *nkx1‐1* is required for the formation of circular fibres. *myoD* RNAi planarians cannot regenerate, while *nkx1‐1* RNAi will cause midline bifurcation.

Both regenerated cells and positional information are required during regeneration and tissue turnover. The positional information is required for tissue patterning and predominately expressed in planarian muscles involving the control of body contractions.^60^ Muscle tissues in planarian are consist of three subtypes of fibres, circular fibres, longitudinal fibres and diagonal fibres (Figure 4). Circular fibres are in the outermost layer and distributed along the medial–lateral axis beneath the epidermis. Just below the circular fibres, diagonal and thin longitudinal muscle fibres network can be found.^59^, ^76^ During the initial regeneration process, PCGs expression in muscles are rapidly activated with ML patterns under sagittal amputation. Meanwhile co‐expression of anteriorly and posteriorly expressed PCGs could be clearly detected in the tail of the planarian after amputated. The production of PCGs does not require neoblasts, and the regenerating muscle could re‐establishing the PCG‐expression domains after injury can occur in the existing muscle cells after injury.^60^ Additionally, planarian muscle can function as the major source of core extracellular matrix (ECM) components, In body wall muscle cells, *Smed‐hemicentin‐1* (*hmcn‐1*) is specifically expressed and encodes a glycoprotein or receptor that interacts with the extracellular matrix, which specifically expressed in body wall muscle cells. *hmcn‐1* RNAi caused a defective regeneration on the planaria head and eye, and causes severe epidermal ruffling. This indicates that *hmcn‐1* plays an important role in the maintenance of localization of multiple parenchymal cells.^76^ Scimone et al. also found that *myoD* is required for the formation of longitudinal fibres arranged along the anterior–posterior axis. After knockdown of *myoD*, planarians became longer and thinner with the lost ability of muscle regeneration (Figure 4). Immunofluorescence experiments demonstrated that upon *myoD* knockdown, longitudinal fibres were lost whereas circular and diagonal fibres remained normal. Planarians without longitudinal fibres could regenerate new tissues, instead of amputated tissues, owing to the defective initiation of regeneration and complete failure of regeneration.^8^, ^59^ Another transcription factor encoding the gene *nkx1‐1* was found to be required for the formation of circular fibres. Eliminating *myoD* or *nkx1‐1* will lead to muscle loss and disruption of body integrity consequently^59^ (Figure 4).

### 7.5 Epidermal

**FIGURE 5:**
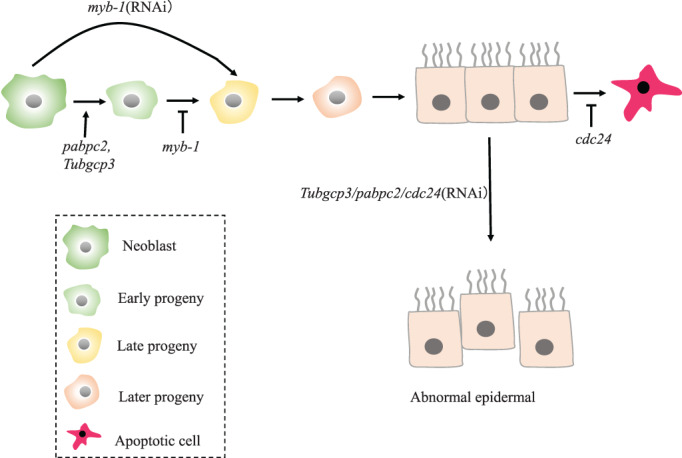
*pabpc2/cdc24* RNAi will result in the abnormal of epidermal. *myb‐1* regulates the specification of the first post‐mitotic progenitor phase, and *cdc42* is required for the maintenance of epidermal lineage.

Epidermal is regulated by a novel cytoplasmic polyA‐binding protein‐PABPC2, which is mainly expressed in epidermal, gut and neoblasts of planarian. Inhibition of *pabpc2* results in the failure of regeneration after amputation. Meanwhile, the uncut planarians with *pabpc2* knockdown developed blastema within 5–6 days post‐2nd feed and further underwent lysis by day 11. Further analysis revealed that *babpc2* knockdown leads to the defects in epidermal lineage specifications, tissue disorders of epidermal and ECM, and dysregulation of wound healing. Proliferation of new cells near the wound is seriously affected. Polysome profiling suggests that epidermal lineages, including *zfp‐1*, are regulated by *pabpc2* at translational level. Those results revealed the role of *pabpc2* in maintaining epidermal and ECM integrity, which is critical for wound healing and subsequent regeneration processes. Whole‐mount in situ hybridization on *pabpc2* knockdown animals at 3 dpa (days post amputation) showed a complete absence of PCG expression both in the anterior and posterior regenerating tissue (blastema). Thus, knockdown of *zfp‐1* gene and transcriptome sequencing analysis proved that epidermal tissue destruction did not affect the expression of PCG, and the translation of PCG transcripts did not require *pabpc2* ^77^ (Figure 5).

*cdc42* (Cell Division Cycle 42), as the most widely characterized member of Rho GTPases, plays a crucial role in the organization and assembly of actin and microtubule cytoskeletons. Report from Zhang et al. showed that *cdc42* is necessary for the maintenance of epidermal lineage. They found that *cdc42* RNAi induced a sustained increase in cell death leading to loss of mature epidermal cells, while the cell division was not affected (Figure 5). Further studies showed that cdc42 inhibited excessive apoptotic cell death during epidermal regeneration and homeostasis of planarians, therefore, *cdc42* is necessary for the maintenance of epidermal lineage.^78^

Pearson et al. used flow cytometry to sort out epidermal progenitor cells for transcriptional analysis and RNAi screening to identify regulatory factors of epidermal differentiation. They identified a myb‐type transcriptional factor (*Smed‐myb‐1*), which is required for the first temporal phase of post‐mitotic maturation. *myb‐1* knockdown inhibits the early stage of differentiation of progenitor cells, but did not affect subsequent epidermal progenitor states or homeostatic turnover and regeneration of the epidermis (Figure 5). These results above indicate the spatiotemporal shift in lineage progression after RNAi of *myb‐1*, and clarify its regulatory role of *myb‐1* in the differentiation process of early epidermal progenitors.^79^ Also, *Tubgcp3* is a homologue gene in planarian *Dugesia japonica*, which regulates the planarian epidermal progenitor cells differentiation. Knockdown of *Tubgcp3* will result in lysis of amputated planarians. In the homeostatic *Tubgcp3*(RNAi) planarians, darker region of epidermis will appear and lead to body lysis in the end.^80^

### 7.6 Intestine

**FIGURE 6:**
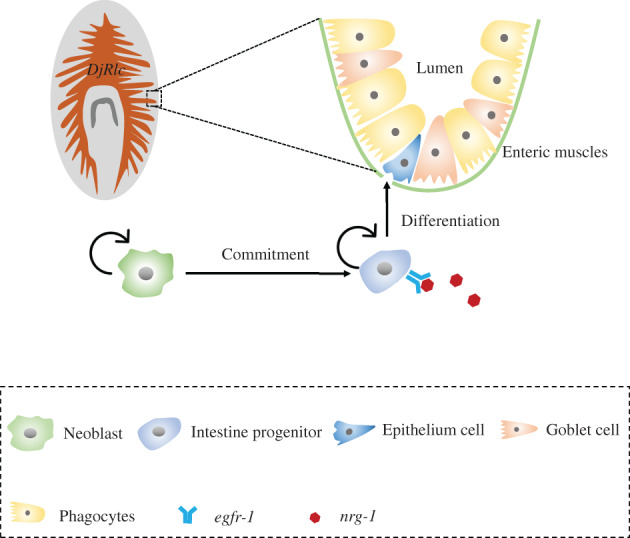
The EGFR signalling pathway controls gut progenitor differentiation during planarian regeneration and homeostasis. Intestine branching morphogenesis is achieved primarily by remodelling of differentiated intestinal tissues.

As the digestive organ of planarians, the intestine can be fully regenerated after amputation. In the process of intestinal regeneration, the new intestinal branches are completely regenerated along with the AP axis, and the intestinal cells are regenerated throughout the whole intestine, instead of specific regeneration sites. Therefore, the formation of the morphology of intestinal branches is mainly achieved through the remodelling of intestinal tissues. As for amputated planarians, existing intestinal tissues contribute to the newly formed intestine. Since intestine cells did not possess dividing ability, so neoblasts will differentiate into intestinal epithelial cells, then the intestinal epithelial cells further transform into other cell types. There is no obvious up‐regulation of the number of proliferative cells in the process of intestinal regeneration, indicating that intestinal regeneration is benefit from the remodelling of differentiated tissues^81^ (Figure 6).

Barberan et al. found that *egfr‐1* in the EGFR family regulates the differentiation of intestinal progenitor cells. After *egfr‐1* RNAi, they found that there was no significant difference between the knockdown group and the control group. However, 4 weeks later, the knockdown group showed fewer gut branches, and the amputated planarians were unable to regenerate intestinal branches. Further immunofluorescence staining showed that the number of intestinal progenitor cells increased after *egfr‐1* RNAi (Figure 6), indicating that the intestinal progenitor cells could not differentiate, resulting in abnormal morphology of intestine.^75^ Recently, a homologue gene of Rlc, *DjRlc* is reported being vital to intestine morphology as well. *DjRlc* is mainly expressed in the intestine of planarians, and RNA interference of *DjRlc* causes defects in morphogenesis of intestine branches in planarians. After amputation, *DjRlc* RNAi delayed the planarians regeneration process of intestine, suggesting that *DjRlc* is required for intestine morphology and regeneration.^82^

### 7.7 Other regulatory mechanisms of regeneration

The regeneration of planarians is a highly complex and coordinated process, involving the regulation of many cellular signalling pathways essential for normal physiological functions during regeneration process, such as asymmetric division, cell cycle, apoptosis, tissue/organ size and polarity determinations. Recently, Arnold et al. found that the number of progeny and the frequency of fission initiation correlate with parent size. By RNAi screening for genes that regulate the size and number of planarian fission, they uncovered a role for TGFβ and Wnt signalling pathways in regulating scale‐dependent behaviour, and the frequency of fission initiation.^83^

Regulation of the cell cycle is also crucial for planarian regeneration. The cell cycle is regulated by many pathways in planarians, such as the Hedgehog signalling pathway and the Hippo pathway. In planarians, Hippo pathway plays an important role in the cell cycle regulation. Early studies have shown that Hippo plays an important role in the development of tissue and organ size. Similarly, disruption of Hippo pathway in planarians could decrease the apoptotic cell death, and induces cell cycle arrest leading to promoted differentiation of post‐mitotic cells. Hippo RNAi resulted in extensive undifferentiated tissue areas and overgrowths, but the body size or cell number are not affected.^84^, ^85^, ^86^ The ERK pathway regulates the differentiation of stem cells during planarians regeneration. Inhibition of ERK pathway prevents the phosphorylation of downstream components and blocked downstream signals. Inhibition of the components of ERK pathway can result in abnormal formation of the blastema and incomplete regeneration of planarian.^29^ In addition, Akt signalling regulates the maintenance and differentiation of planarians stem cells. Abrogating *Smed‐Akt* with RNAi can reduce the number of stem cells of the planarian while intensified the cell death, thereby impairing the function of neuron and excretory systems. At the same time, cell death will be intensified. Although the stem cells still have the stress response to injury after knockdown, normal blastema cannot be formed and regeneration cannot be completed eventually. In summary, Akt signalling is critical in organismal physiology, as well as regulates neoblast biology.^87^ Rink et al. found that the tail part of the Planarian *Dendrocoelum lacteum* did not have regeneration ability after head removal, but after eliminating the Wnt signal pathway *Dlac‐β‐catenin‐1*, functional regeneration of tail pieces was fully restored. These results indicate that Wnt not only plays an important role in the A/P axis of planarians, but also is crucial for the regeneration ability of planarians.^88^

## 8 PROSPECTIVE

In this review, we mainly summarize the recent progress about the molecular mechanisms for regulating planarian regeneration, and discuss the molecular signalling pathways that are regulated during regeneration in tissues, in particular the regulation of neoblasts in tissue regeneration and the mechanisms behind the tissue morphology maintenance. Here we draw a picture of planarian regeneration in tissue level, and summarize the factors during the tissue regeneration process in recent years. Planarian regeneration starts with differentiation and self‐renew of cNeoblasts. The cNeoblasts will first differentiate into tissue progenitors under the regulation of genes such as *egfr‐3*, which will form the blastema. The sense of injuries and initiation of regeneration is regulated by diverse genes. Finally, the neoblast within the blastema differentiate into desired cell types and regenerate the missing tissues, which direct by signalling pathways and bioelectric signals. We summarize the recent mechanisms in tissues as following:

1. Eye spot

Regeneration of eye spot start with the differentiation of eye progenitor cells. *ovo* and *sp6‐9* specifically express in eye progenitor cells. Regeneration process is regulated by *egfr‐4*, *Smed‐smad6/7‐1* and *Smed‐smad6/7‐2*. *notum* directs the position of axon. Guidepost‐like cell, existing outside of visual system and acts like a landmark to guide the location of eye's regeneration.

2. Central nervous system

Ectodermal lineage progenitors can differentiate into epidermal and neuronal cells in the regulation of *soxB1*. During the regeneration process, *soxB1*, *lhx1/5‐1*, *pitx*, *egfr‐4* and so on are important. *pitx*, *slit*, *wnt1* and *wnt11‐2* will construct the midline of CNS.

3. Excretory system

The maintenance and regeneration of the planarian excretory system are mainly regulated by EGFR signalling.

4. Muscle

In the regeneration of process of muscle, *myoD* is required for the formation of longitudinal fibres, while *nkx1‐1* is required for the formation of circular fibres. *myoD* RNAi planarians cannot regenerate, while *nkx1‐1* RNAi will cause midline bifurcation.

5. Epidermal

During epidermal regeneration, *myb‐1* plays an important role in the specification of the first post‐mitotic progenitor phase of epidermal. *pabpc2/cdc24* RNAi will result in the abnormal of epidermal.

6. Intestine

The EGFR signalling pathway controls gut progenitor differentiation during planarian regeneration and homeostasis. Intestine branching morphogenesis is achieved primarily by remodelling of differentiated intestinal tissues.

These molecular mechanisms together can give us an overview of regeneration process in every tissue. Neoblasts differentiate into tissue progenitor cells. The regeneration polarity is determined by Wnt pathway, BMP pathway and bioelectric signals. Then those tissue specific genes will regulate the tissue progenitor cells differentiating into desired cell types. The environmental factors will participate the regeneration process.

Even there are countless work trying to reveal the explicit network underlying the regeneration process, still there are a lot questions to be resolved. We cannot distinct the cNeoblasts with other subtype neoblasts, and there is limited knowledge about the molecular signature in neoblast during their differentiation. We can learn that Wnt pathway, Bmp pathway, Ft/Ds pathway and bioelectric signals will control the polarity of planarian from previous works, but how the polarity in large‐scale shapes coordinated with cell‐level properties? Many genes will control the regeneration of tissues, still we do not know how does regeneration know when to stop? And there is little researches on the morphology control of regeneration. We hope more and more researchers will be interested in these questions and focus on the field and make enormous contribution.

Planarian provides a valuable model system to dissect the underlying mechanisms for cell differentiation and tissue/organ regeneration in mammalian. Based on this, accumulating data about the regulatory molecular events have been uncovered and many regeneration models have been proposed. However, the detailed molecular mechanisms for planarian regeneration are still unclear, such as how the newly differentiated cells are directed to the wound site, and what are the underlying molecular network in coordinate regulation of neoblast differentiation. Since then, researchers have developed lots of approaches to investigate the regeneration. With the development of technology, we will have a clear understanding of the regeneration mechanism of the planarian, such as in suit hybridization,^15^ RNAi,^89^ single cell sequencing and other technology.^16^, ^90^ Though we can routinely use RNAi to reduce the expression of desired genes, there is no way to knockout the genes. Single cell technology is becoming more and more common, and the cost is gradually reduced. Moreover, the newly developed spatial transcriptome technology can be used at the same time. Spatial transcriptome can not only explore the detailed transcriptome information during regeneration, but also the spatial information, which will help us to understand the spatial information during regeneration. Levin et al. based on biochemical and biomechanical approaches, providing a brand new physical insight and useful biochemical approaches. The directed self‐assembly of multicellular processes requires the understanding and regulation of the emerging structures, so this is of great significant for synthetic morphology.^91^ And the computationally modelling of planarian regeneration is rapidly developing, which will provide new angles to understand the planarian regeneration. The comprehensive use of multiple technologies is of great help to solve the mysteries on planarian regeneration.^92^

The recent advances in the RNA methylation have provided possible epigenetic mechanisms for the regeneration process. For instance, m^6^A play an important role in the maintenance and development of haematopoietic stem cells regeneration in vertebrate,^93^, ^94^, ^95^ pointing to its essential roles in the process of tissue regeneration.

Although, there are a variety of databases providing the genome and transcriptome information needed for analysis, there is no complete genome data available due to high complexity of the genome and the difficulty of assembling. Also, the annotation information of the planarian is incomplete, and many specific genes of the planarian has not been defined by functional characterization. These genes are likely to play crucial roles in the regeneration process, but functional validation of these genes needs the joint efforts of many laboratories. At present, more and more technical means have been developed to analyse biological problems, which will be of great help to the study of the regeneration of the planarian. It is highly expected that, with the development of computing resources and technical approaches, the regeneration mechanism for the planarian will be eventually clarified.

## AUTHOR CONTRIBUTIONS

**XYG** collected the related literature and drafted the manuscript. **GSC**, **XH** and **YLZ** participated in the design of the review and drafted the manuscript. **YGY** conceived the concept and supervised this manuscrpt. All authors read and approved the final manuscript.

## Data Availability

I confirm that my article contains a Data Availability Statement even if no data is available (list of sample statements) unless my article type does not require one. I confirm that I have included a citation for available data in my references section, unless my article type is exempt.
